# The visual and semantic features that predict object memory: Concept property norms for 1,000 object images

**DOI:** 10.3758/s13421-020-01130-5

**Published:** 2021-01-19

**Authors:** Mariam Hovhannisyan, Alex Clarke, Benjamin R. Geib, Rosalie Cicchinelli, Zachary Monge, Tory Worth, Amanda Szymanski, Roberto Cabeza, Simon W. Davis

**Affiliations:** 1 Center for Cognitive Neuroscience, Duke University, Durham, NC USA; 2 Department of Neurology, Duke University, Durham, NC USA; 3 Department of Psychology, University of Cambridge, Cambridge, UK; 4 Department of Psychology & Neuroscience, Duke University, Durham, NC USA

**Keywords:** Object recognition, Semantic memory, Concepts, Memory

## Abstract

Humans have a remarkable fidelity for visual long-term memory, and yet the composition of these memories is a longstanding debate in cognitive psychology. While much of the work on long-term memory has focused on processes associated with successful encoding and retrieval, more recent work on visual object recognition has developed a focus on the memorability of specific visual stimuli. Such work is engendering a view of object representation as a hierarchical movement from low-level visual representations to higher level categorical organization of conceptual representations. However, studies on object recognition often fail to account for how these high- and low-level features interact to promote distinct forms of memory. Here, we use both visual and semantic factors to investigate their relative contributions to two different forms of memory of everyday objects. We first collected normative visual and semantic feature information on 1,000 object images. We then conducted a memory study where we presented these same images during encoding (picture target) on Day 1, and then either a Lexical (lexical cue) or Visual (picture cue) memory test on Day 2. Our findings indicate that: (1) higher level visual factors (via DNNs) and semantic factors (via feature-based statistics) make independent contributions to object memory, (2) semantic information contributes to both true and false memory performance, and (3) factors that predict object memory depend on the type of memory being tested. These findings help to provide a more complete picture of what factors influence object memorability. These data are available online upon publication as a public resource.

## Introduction

One of the most important issues in memory research is why we remember some things but forget others. To address this issue, it is critical to answer not only which *processes* lead to successful encoding (e.g., depth of encoding effects; see Craik & Tulving, 1975) and/or retrieval (e.g., transfer-appropriate processing; see Morris et al., 1977), but also what *contents* of these events are more memorable than others. Although these two questions are closely related, their focus is different: the former concentrates on the actions of the person remembering, and the latter on properties of the stimuli. While the *processes* question has been a continuous focus in memory research since its inception, the second question has received much less attention. However, the question of which stimulus properties are easier to remember has been rapidly growing in popularity in recent years. Studies that focused on the concept of intrinsic memorability have typically used a combination of visual factors to predict subsequent memory of scenes (Bainbridge et al., 2017; Isola et al., 2014), objects (Jaegle et al., 2019), and unfamiliar faces (Bainbridge et al., 2013). Very few such memorability studies have examined verbal stimuli, and the semantic factors examined in such studies are usually limited to category membership (Bainbridge & Rissman, 2018) or automatic labels generated by automated computer vision algorithms (Borkin et al., 2016; Isola et al., 2014). More importantly, no study – to our knowledge – has simultaneously examined both visual and semantic factors, which is essential to understand memory for everyday scenes and objects. This was our overarching aim.

The current study relates two different but interconnected literatures. First, we relate findings in the neuroscience (Grill-Spector & Malach, 2004) and behavioral (Pylyshyn, 1999; Rosch et al., 1976) visual perception literatures that are relevant to explaining the memorability of visual stimuli. For example, memorability studies have shown that, despite a high human capacity to remember visual details (Brady et al., 2008), simple image measures, such as pixel statistics, image complexity, or the number of objects in an image, do not predict how well individual objects are remembered (Isola et al., 2014). One possible explanation for null findings is that simple image measures do not match the way the visual cortex processes visual information. To investigate this idea, the current study examined how memory for objects is predicted by measures of visual processing provided by a *deep neural network* (DNN). DNNs model visual processing in primate visual cortex using convolutional layers (Kriegeskorte, 2015). Although DNNs were originally designed for image classification in computer vision, they have been shown to be excellent neuroscience models for visual processing (Rajalingham et al., 2018; Yamins et al., 2014), often surpassing traditional theoretical models (e.g., HMAX, object-based models; Cadieu et al., 2014; Groen et al., 2018). In the current study, a DNN yielded measures of visual object processing that were used to predict subsequent object memory.

Second, the current study relates to findings in the semantic cognition literature that are relevant to explaining object memorability. Most memory studies examining the influence of semantic factors rarely incorporate visual features as predictors of memory strength and have centered on verbal stimuli and simple lexical factors like word frequency or concreteness (e.g., words that reflect more concrete concepts tend to be remembered better; see Fliessbach et al., 2006). Research on semantic factors in memory for objects is very scarce; including a few studies with neuropsychological patients (Kraut et al., 2002; Patterson, 2007), some studies on how labeling enhances or distorts memory for objects (Koutstaal et al., 2003; Richler et al., 2011; Richler et al., 2013) , and a limited number of studies on basic conceptual properties, such as the nameability (Richler et al., 2013) and typicality (Qin et al., 2014). However, there is virtually no evidence on how complex conceptual statistics determine object memorability. This question can now be examined by taking advantage of the *conceptual structure account* (CSA), which provides a comprehensive framework to quantify and formalize the relationship between semantic and visual features of objects in terms of their distinctiveness and interrelatedness (Devereux et al., 2018; Moss et al., 2005; Taylor et al., 2012). In the current study, the CSA provides measures of semantic properties of objects and is used to predict subsequent memory for object images.

In sum, the current object memory study investigated how well visual and semantic properties predict the visual and lexical memory of object concepts. Before this experimental study, it was necessary to conduct a normative study of the visual and semantic features of a large set of everyday object images. Available published norms comprising semantic properties for object concepts are only available for words (Devereux et al., 2014; Ken McRae et al., 2005), but currently there is no normative concept feature data on object images. In the current semantic norming study, each participant provided semantic features for a small set of different object images. Creating these norms was a prerequisite step for the study, but it was also a goal in itself, because norms of the visual and semantic features of a large set of objects is critical for functional magnetic resonance imaging (MRI) and memorability studies with objects. Our norms are freely available online (http://mariamh.shinyapps.io/dinolabobjects), along with regularized copies of the images.

In the memory study, which consisted of two experiments, visual and semantic variables were used to predict subsequent memory for objects. Complex visual measures were obtained by analyzing the object pictures using the layer-specific activation information from a popular DNN, AlexNet (Krizhevsky et al., 2012), and feature-based semantic metrics (*mean distinctiveness* and *correlational strength*) were obtained by an analysis of concept feature norms. We also examined more basic visual (e.g., basic pixel statistics) and semantic (e.g., word frequency) metrics. In each of the two memory experiments, every item in the corpus was tested in a *visual memory test* (Day 1: picture target; Day 2: picture cue) and a *lexical memory test* (Day 1: picture target; Day 2: lexical cue). We use these two different memory tasks to evaluate the conceptual and perceptual properties of the object images. We conducted the visual memory test to investigate the resiliency of memory across exemplars and the lexical memory test to investigate the contribution of semantic information to the memorability of object images. Both tests allow us to examine the contribution of visual and semantic properties in perceptual and conceptual memory. These tests are important for understanding (1) why some properties might contribute more to memory in one test than another, (2) if complex visual and/or semantic information is important regardless of the memory test, and (3) whether semantic or visual information, either simple or complex, contributes to memory when tested in the same domain (e.g., semantic information contributing more to memory in the lexico-semantic task than in the visual task).

## Visuo-semantic object-norming study

### Methods

#### Participants

Five hundred and sixty-six Amazon Mechanical Turk workers all with a 95% approval rating or above (347 females, 19–75 years of age, mean age = 34.6 years, all self-reported native speakers of American English) participated in this study. Participants had an average of 14.68 years of education, and the racial demographic was balanced with national averages. Participants could take part in repeat sessions and completed between one and five sessions. Sessions lasted about an hour with 40 concepts presented per session. Participants were paid $3.00 for their participation in the property norming study. Informed consent was obtained from all participants under a protocol approved by the Duke Medical School Institutional Review Board (IRB). All procedures and analyses were performed in accordance with IRB guidelines and regulations for experimental testing.

#### Materials

The two primary aims of this study were to (1) collect normative feature data on a large set of object concepts that can be expanded and manipulated by a wide range of research domains and 2) assess whether feature statistics can explain memorability of objects. To date, the most extensive and widely used set of property norms are the McCrae Norms (Ken McRae et al., 2005) and the Centre for Speech, Language and the Brain (CSLB) norms (Devereux et al., 2014), both of which are considered to be the standard for semantic feature representations of concepts. Both norms provide information on type of features and feature production frequency for a large number of concrete objects. However, both databases are based on responses to verbally presented stimuli, and therefore may not directly inform the memory for visual object stimuli. Nonetheless, throughout this paper, we use both CLSB norms and the McCrae Norms as a guide to characterize our dataset.

##### Stimuli

**Fig. 1:**
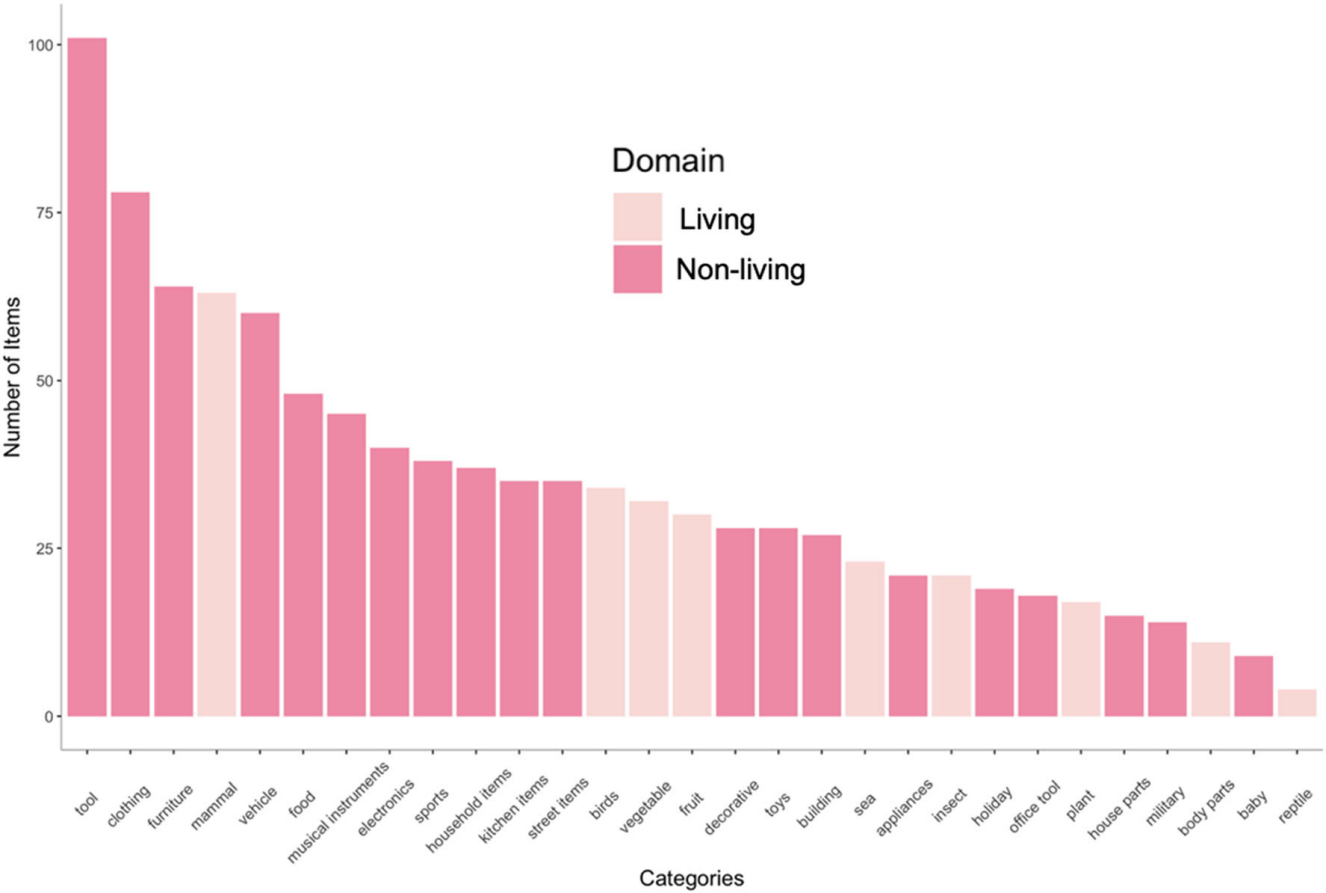
Distribution of Item Frequencies across different Categories. The bar chart represents the number of items in each category in our dataset, split by living (pink) and nonliving (dark pink) categories

A total of 995 object concepts were used for the online object norming via Amazon Mechanical Turk (AMT). Image concepts were selected from a wide range of standard object categories (e.g., birds, buildings, mammals, tools, vehicles), as well as object categories present in everyday life, but not well represented in typical object databases (food, holiday items, street items). 237 of the objects were living and 758 were non-living. The relative size of each of the 29 categories in our database is depicted in Fig. 1. In selecting these concepts, we included concepts from CSLB norms that were familiar to English speakers, as well as additional object concepts to help balance the number of items across categories. We aimed to avoid ambiguous concepts and only included concepts in our analyses that had three or more features. We organized our 995 object concepts under a range of standard categories (Fig. 1); as is typical of such naturalistic object datasets, non-living categories outnumber living categories by about 2 to 1, and tools constitute the largest category of items.

Suitable images for each concept were selected from the image search engines Google Images, Bing Images, and Flickr. Images were selected based on the following criteria: (1) minimum size of 300 x 300 pixels; (2) either whitespace background, a background easily removable with image-editing software, or a background not otherwise integrated into the foreground or target object; (3) standard framing/positioning of the object, i.e., we avoided image orientations that obscured the identity of the object; (4) all images were in color, with no obvious chromatic or morphological filter; (5) no visible watermarks; and (6) no text printed on the object concept identifying it as such (e.g., “Fire Station No. 9”). After assembling two image exemplars for each concept, backgrounds were removed with photo-editing software and images were cropped to square dimensions and resized to 300 x 300 pixels.

#### Image attributes

**Fig. 2:**
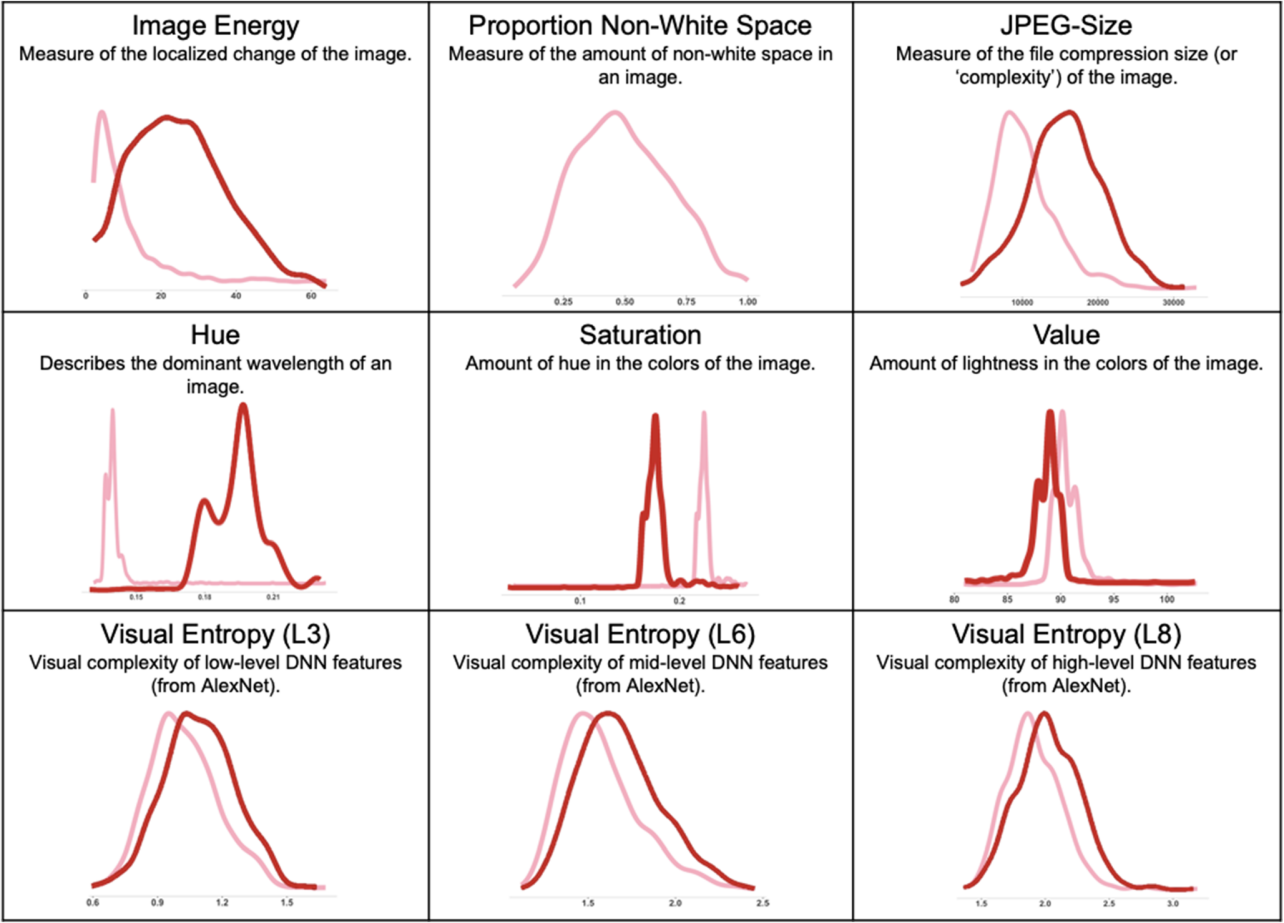
Visual measures used in the current study. Note that original scores (pink line) have been adjusted with log transformations (dark line) if and only if they did not meet standards for normality. If a measure lacks a dark line such a transformation was not needed

Image attributes in the current analysis are characterized both as intrinsic properties of object identity and as potential predictors of object memorability. Attribute definitions, characteristics, and distributions within the current dataset are summarized in Fig. 2 (Visual features) and Fig. 4 (Semantic features). Visual measures comprised basic pixel and image statistics, as well as more complex statistics defined by the entropy of individual layers of a popular convolutional deep neural network (i.e., AlexNet). Semantic measures comprised frequency, as defined by the Corpus of Contemporary American English (COCA), name agreement, and the number of constituent features, as well as more complex statistics defined by the relation between features of items including *mean distinctiveness* (MD), *correlational strength* (CS), and a *correlation x distinctivenes*s measure (CSxD). We describe these measures, including descriptive statistics of the underlying distribution of these measures, in more detail below.

##### Visual features

The first question addressed by the current article is whether simple image features are predictive of memory in the visual and lexical memory task. Visual measures are summarized in Fig. 2, including descriptive statistics on the underlying distribution and, when necessary, correction of the distribution to improve normality. We first calculated a number of low-level image features that describe item-wise values for a given image. Many of these properties have been shown to not be predictive of image memorability (Dubey et al., 2015; Isola et al., 2014), but underlying questions remain about the capacity for these basic visual features to predict conceptual memory. Basic pixel statistics such as Hue, Saturation, and color Value (commonly referred to as “HSV”) were calculated on each image in our database, as well as the proportion of non-white space in the normalized image. Image energy, a measure of the localized change of the image, and JPEG size, an indirect measure for image complexity based on image compression (Torralba & Oliva, 2003), were also included in the analysis.

##### Deep convolutional neural network similarity

**Fig. 3:**
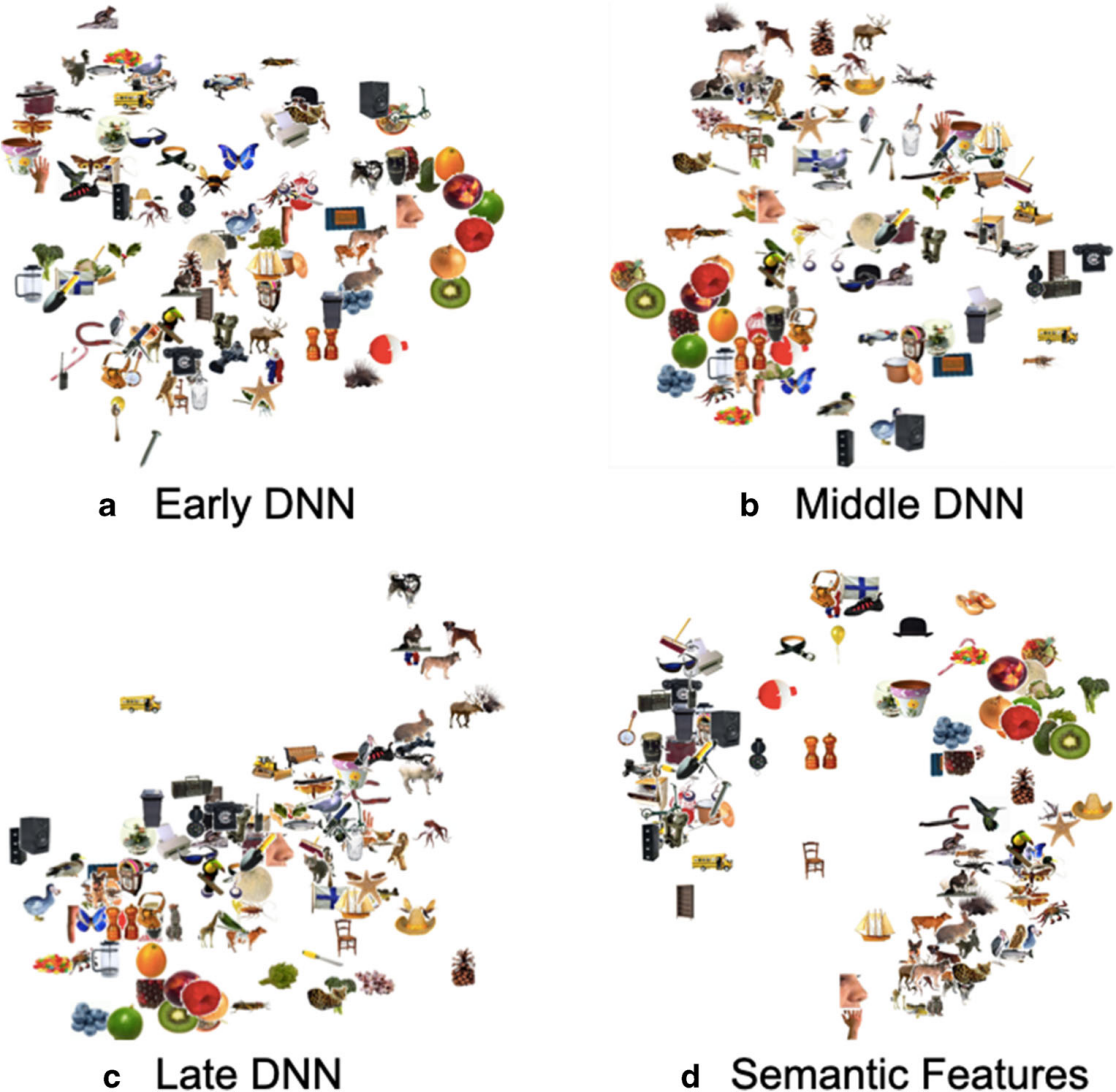
Multidimensional scaling plots for object stimuli. (**a**–**c**) MDS visualization of DNN layers in the current analysis, based on early, middle and late layers of AlexNet; (**d**) MDS of Semantic features, based on normative feature collection. MDS plots presented above are for a subset of the stimuli for easy viewing. The online Shinny App is available for users to zoom in on each figure

Next, we assessed complex visual properties of object images by assessing the similarity of visual features derived from a DNN, which carries inherently relational information given that (1) a DNN optimizes based on all images within a training set, and (2) individual layers represent distinct but still dependent information between layers as image vectors change through the progression across layers (DNNs; Krizhevsky et al., 2012; LeCun et al., 2015). DNNs consist of layers of convolutional filters and can be trained to classify images into categories with a high level of accuracy. During training, DNNs “learn” convolutional filters in the service of classification, where filters from early layers predominately detect lower-level visual features and from late layers, higher-level visual features (Zeiler & Fergus, 2014). Therefore, a DNN is an ideal model to investigate multi-level visual feature distinction. Here, we used AlexNet, which was successfully trained to classify 1.2 million high-resolution images into 1,000 different categories (Krizhevsky et al., 2012). AlexNet consists of eight layers including five convolutional and three fully connected layers. We extracted the activation values for three representative layers (Layers 3, 6, and 8 for early, middle, and late DNN layers, respectively) for each image and converted them into one activation value per object. Multidimensional scaling provides a qualitative illustration of the possible image dimensions. For example, the early visual MDS plot (Layer 3, Fig. 3A) organizes concepts largely by shape (thin, vertically oriented objects are distributed on the left side, with horizontally shaped objects towards the left, and circular objects on the right side). The middle visual MDS plot (Layer 6, Fig. 3B), in contrast, suggests more complex frequency and orientation information, with high visual frequency (items with thin parts or changes in color or luminance) towards the top of the image, and items with low visual frequency information (unitary color and luminance across the item) towards the bottom. The late visual MDS plot (Layer 8, Fig. 3C) retains some of this complex configural information, but also begins to group items of similar categories together in loose categorical clusters (e.g., fruit towards the bottom left, animals towards the top right). Lastly, the MDS plot for semantic feature information groups items in a configuration roughly consistent with their categorical organization. In Fig. 3D, living things are mostly organized on the right side, for example, animals are distributed in the bottom right corner and foods in the top right corner, while non-living things are largely organized on the left side with clothing items distributed towards the top and tools and furniture distributed towards the middle bottom side.

#### Semantic attributes

**Fig. 4:**
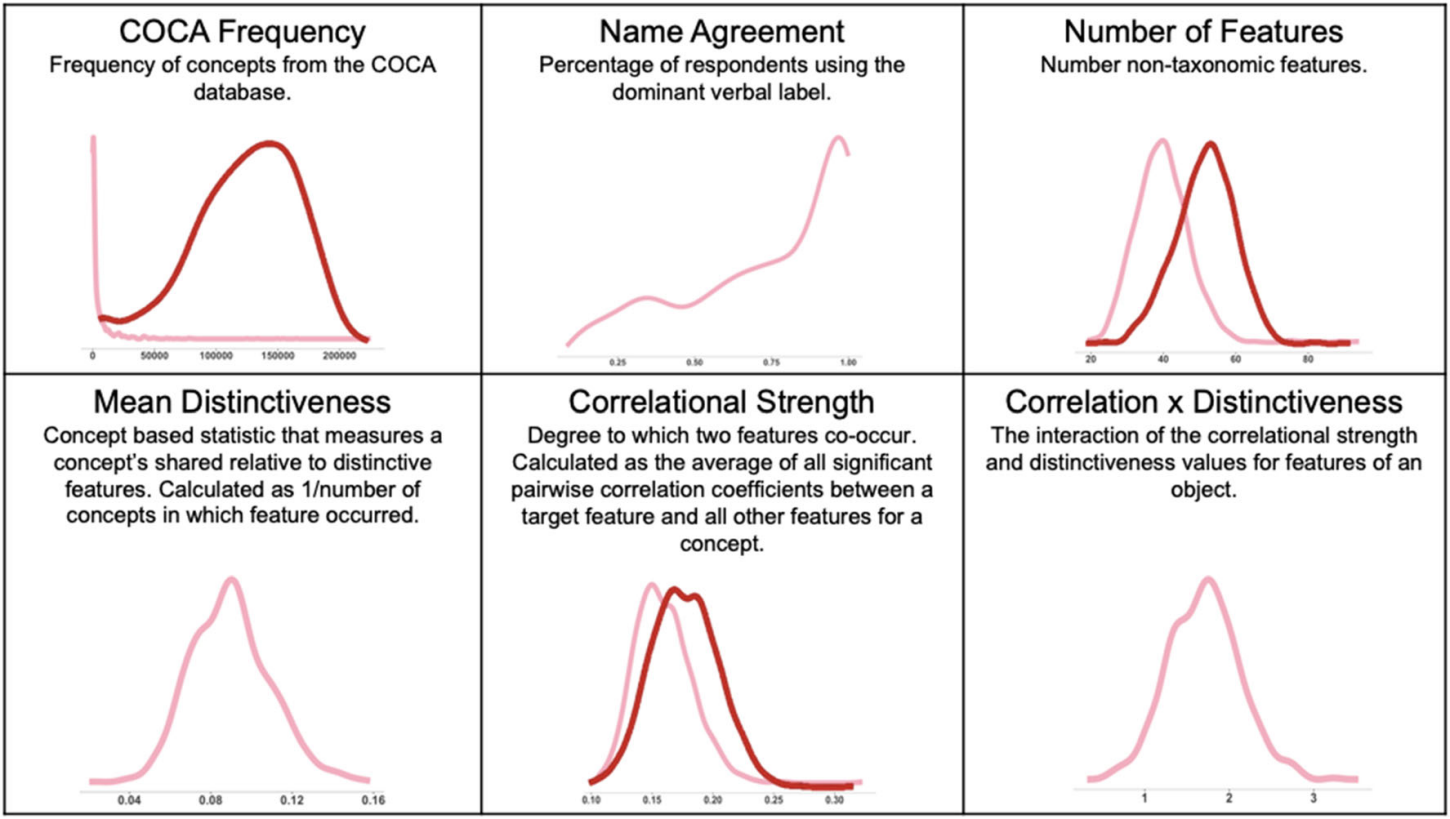
Semantic measures used in the current study. Note that original scores (pink line) have been adjusted with log transformations (dark line) if and only if they did not meet standards for normality. If a measure lacks a dark line such a transformation was not needed

Next, we describe the semantic attributes in the current analysis, as summarized in Fig. 4. First, a number of basic concept features were assessed. Concept (or lemma) frequency was assessed in our sample using the word frequency provided in the Corpus of Contemporary American English (Davies, 2008), which contains 425 million entries sampled from a broad range of written sources.

Name agreement, which reflects the agreement for a verbal label to an object photograph, was assessed with a standard picture-name agreement task (Snodgrass & Vanderwart, 1980). For every image, 25 Duke University undergraduates from introductory psychology courses identified each picture as briefly and unambiguously as possible by writing only one name for each image. Participants were instructed to respond “don’t know” if the picture was an object unknown to them, or if they didn’t know the name. Name agreement was then calculated as the proportion of participants identifying the modal name for a given object photograph. The number of features (NoF) for each object was also calculated and included in our analysis. This metric is a general semantic property that ignores the semantic content of those features; only non-taxonomic features were included when calculating the total number of features for an item. A taxonomic feature indicates superordinate category information to which a concept belongs (Ken McRae et al., 2005).

##### Semantic property norms

The principal approach of the current analysis is the application of a new and large set of property norms designed to characterize the semantic features associated with a broad range of object concepts. While many of the concepts overlap with the McRae (Ken McRae et al., 2005) and CSLB (Devereux et al., 2014) norms, the aim of the current corpus is to offer semantic norms associated not with verbal descriptors, but instead specific object photographs associated with those object concepts. We make these data available on our GitHub site (https://github.com/ElectricDinoLab), allowing researchers to estimate their own cutoff points for production frequencies associated with each object feature or concept.

##### Feature-based statistics

The feature-statistics used in this study are based on the conceptual structure account (CSA), a neurocognitively motivated theory of conceptual knowledge that captures information of conceptual representations (Taylor et al., 2011; Tyler & Moss, 2001). Feature statistics quantify the smaller elements of a concept, the semantic features, which provides a useful metric to assess behavior. Here, we use feature statistics to (1) assess the relational structure between features of items in our dataset and (2) use this structure to assess the dimensionality of memory scores from the memory task. In the current study, feature-based statistics from the CSA are used to characterize the relational structure between semantic features of items.

In addition to the simpler feature statistics described above, the current analysis sought to test the utility of object features in predicting memorability of items in either the lexical or visual memory test. We used three key measures that are capable of differentiating between similar objects. First, *mean distinctiveness* describes whether concepts have more distinct versus shared features. For each feature, a distinctiveness value is calculated by taking 1/number of concepts in which the feature occurred, with mean distinctiveness being the average value across the features in the concept. Non-living things tend to have more distinctive features than living things, owing generally to the fact that living things have more shared features (e.g., eyes, nose, legs) than do non-living things (e.g., tools & vehicles). Concepts that have more distinct features will have fewer semantic neighbors, thus activating a unique conceptual representation (Clarke & Tyler, 2015). Second, *correlational strength* describes how features of a concept co-occur; in other words, correlational strength for a concept is greater for objects composed of highly co-occurring features (e.g., *has legs* and *has feet are often found in the same concept*) that will mutually coactivate, facilitating feature integration and activation of the concept (Clarke & Tyler, 2015; K. McRae et al., 1997). While both mean distinctiveness and correlational strength are measures common to a number of feature-based accounts (e.g., Cree & McRae, 2003; K. McRae et al., 1997; Mirman & Magnuson, 2009; Rogers et al., 2004; Devereux et al., 2016), our third measure, correlational strength and sharedness (*Correlational Strength x Distinctiveness* or *CSxD*) is specific to the CSA. CSxD describes the relationship between the correlational strength and distinctiveness of the features for each concept (Taylor et al., 2012). This measure is defined as the unstandardized slope of the regression line that describes the interaction of these two statistics (Taylor et al., 2012).

#### Procedure

The current feature-norming dataset comprises 995 objects from 29 different categories and includes 5,520 features, each of which was present at least three times in the data. Taxonomic features (e.g., *is a dog* or *is a mammal*, for the example object *dalmatian*) are not typically considered true semantic features and thus were not used in the analysis of this dataset (Devereux et al., 2014; Ken McRae et al., 2005).

**Fig. 5:**
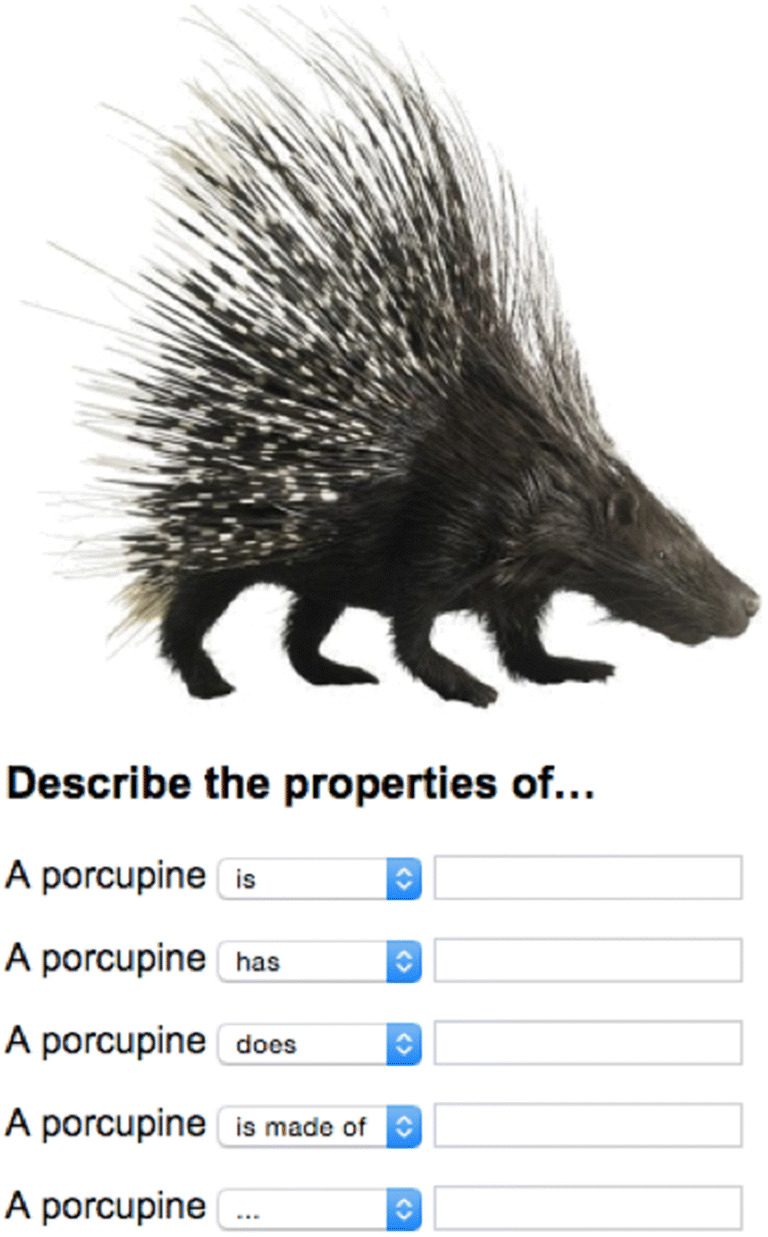
Feature-norming example. Example stimuli and prompt that participants saw during the feature-norming task

Participants were shown an object (e.g., a porcupine) and were given a space to add five unique features; similar to previous feature-norming paradigms (Devereux et al., 2014; Ken McRae et al., 2005), participants were limited to five features, and were prevented from proceeding through the task if all features were not completed. The participants were asked to select a relation word from a drop-down menu, with presets for <is>, <has>, <does>, <is made of>, and “…” (participants were instructed to use the blank space as they wished to specify some other relationship). The default pull-down option for the five feature-response cues was set to one of each of the above five options, as presented in Fig. 5. Participants could use any of the pulldown verb options, they were not required to use all of them, but were required to provide five features for each concept. Concepts were randomized so that two concepts from the same category did not appear consecutively and that each concept was presented to at least 20 participants. Each participant was presented with a series of 40 objects, presented pseudorandomly across participants, with an even distribution of objects per category. Participants were allowed to complete between one and five Human Intelligence Tests (HITs) of the feature norming task. AMT workers were prevented from performing the same HIT twice, and each set of 40 items comprised a unique subset of objects; thus, no objects were repeated for participants who completed more than one feature-norming HIT. In order to receive full credit for their participation, workers needed to complete all five spaces. Data from 566 total participants (with an average of 30.5 participants contributing to each concept) were eventually used to create a feature x concept production frequency matrix.

Before the construction of the production frequency matrix, feature responses underwent various stages of processing, following the procedures used by McRae et al. (2005) and Devereux et al. (2014). These steps, done by hand, included: (1) removal of adverbs, such as *really* and *very*, (2) feature-splitting, for example a feature such as “*has a round face*” was rewritten as “*has a round face*” and “*has a face,*” (3) synonym mapping, which involves identifying synonyms both within and across each concept; for example “*does travel in groups”* and “*does travel in packs”* and “*does travel in a flock”* were collapsed to “*does travel in groups,”* (4) correction of spelling mistakes and when incorrect relation words were used (e.g. “has a luxury item”) were also changed when the meaning was clear (“is a luxury item”) , (5) morphological mapping, for example “*is used in cooking*” and “*is used by cooks*” were collapsed together as “*is used in cooking,*” (6) removal of plural forms, and (7) removal of features not present in at least two concepts. Relation words were not changed. At all stages of processing the data, results were checked manually and were corrected if necessary to prevent from excessively modifying the features and to maintain inter-rater reliability. After preprocessing, a feature x concept production frequency matrix was created to describe the normalized frequency of a given feature for a given concept. The resulting preprocessed features were then collected into various feature-label groups, and summary statistics were calculated on all features. An example for a subset of features, their relation word, and production frequency for the object *bee*, are found in Table 1, which depicts both the individual features as well as the production frequency (i.e., the number of times that feature was mentioned across all raters) of each feature. We used a production frequency cut-off of three, such that only features that occurred at least three times were used in the analysis of the dataset.

**Table 1:** Example concept properties

Object	Relation word	Feature	Production frequency
	Has	wings	21
	Is	an insect	13
	Does	make honey	12
	Does	sting	11
	Has	a stinger	11
	Does	fly	10
	Is	black	8
	Is	yellow	8
	Has	legs	7
	Does	pollinate flowers	6

## Memory studies

### Methods

#### Participants

A group of 200 Amazon Mechanical Turk workers (>95% approval rating in AMT, all self-reported speaker of native English) participated in the lexical memory task and a different group of 303 workers participated in the visual memory task. Both groups of participants were different than the group of participants who completed the normative study. Forty people were excluded from the visual memory task and seven people from the lexical memory task, because of a computer error that did not collect their responses.

A total of 456 Amazon Mechanical Turk workers completed either the visual (n = 263) or lexical (n = 193) memory task. In the lexical memory task, participant ages ranged from 19 to 87 years, mean age = 39.7 years, with 108 females and 85 males, and in the visual memory task ages ranged from 18 to 76 years, mean age = 37.1 years, with 137 females and 126 males. The demographics in the two memory tasks are comparable, such that there are no significant differences in sex t(454) = -0.93, p = 0.35, years of education t(399) = -1.10, p = 0.27, race t(445) = 0.18, p = 0.86, and lag, which is the difference between the time when the participant completed encoding and retrieval, t(245) = 1.10, p = 0.27. There is a significant difference in age, t(406) = 2.11, p = 0.04, between visual and lexical memory groups, but this factor was not a significant predictor of either visual (r = 0.07, p = 0.32) or lexical memory (r = 0.07, p = 0.31).

#### Procedure

**Fig. 6:**
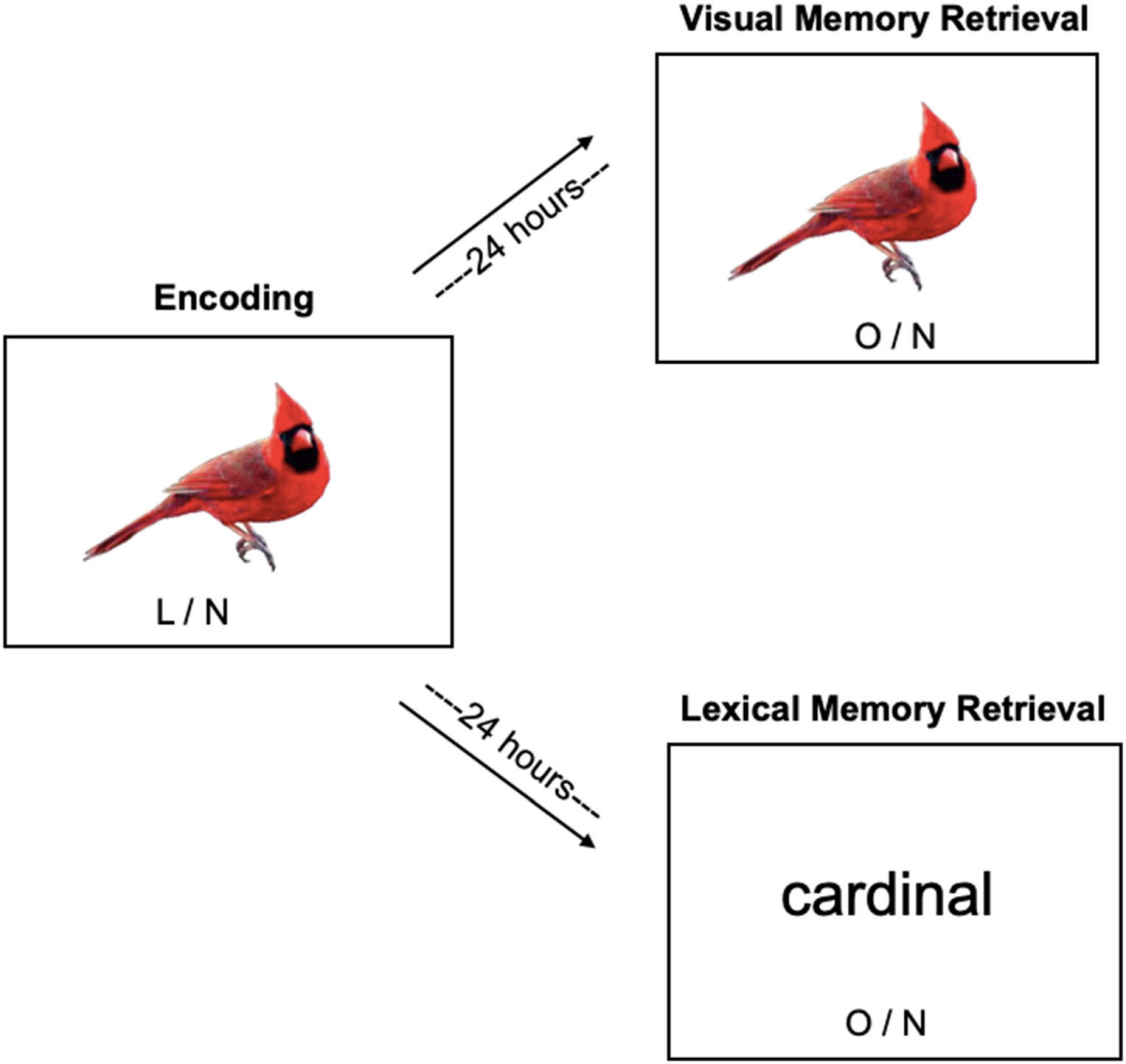
Memory study paradigm. Example stimuli during the visual and lexical memory task. Both tasks comprinsed of an Encoding session where visual object stimuli were presented; subsequent Retrieval Sessions presented old and new objects (visual memory task) or word stimuli (lexical memory task)

AMT workers were presented with either a visual or a lexical memory test (Fig. 6). Materials used in both Visual and Lexical memory studies were the 995 object concepts from the normative study. In both tasks, AMT workers were presented with the same encoding task, and not informed of the subsequent retrieval session to be completed afterwards (i.e., incidental encoding). Participants were presented with a series of object images during the Day 1 HIT, during which they identified whether the object was living or non-living; each of 168 objects was presented for 2 s with a 1-s inter-trial interval. On the Day 2 Retrieval session, 24 h (±4 h; mean lag between Encoding and Retrieval = 29.34 h) after the Encoding HIT, participants were presented with either a Visual task (shown object images) or a Lexical task (shown words representing objects), with 168 old stimuli intermixed with 168 new stimuli. In both Encoding and Retrieval HITs, objects (old and new) were balanced across object categories and balanced for COCA frequency across all image sets. In both retrieval sessions, participants made a 2-alternative forced-choice oldness decision. Total run time for these Encoding and Retrieval HITs were approximately 15/30 min, respectively. Workers were paid $0.50 for completing the Day 1 encoding session (mean time to finish 16.07 minutes), and $4.50 for completing the Day 2 retrieval session (mean time to finish for both the picture and word tasks was 23.5 min).

After data collection, mean hit rates and false alarm rates for each item were calculated based on the percentage of correct responses across subjects to old or new trials, respectively. As such, each measure represents an item-wise averaging across the responses of all contributing AMT workers, and is therefore expressed as a continuous measure amenable to linear regression. As analyses were focused on the item level, each item was presented *only* as a new or an old item. In total, memorability information from 456 participants was used to establish an individual memory score (Visual or Lexical memory, Hit Rate and False Alarm Rate). An average of 43 AMT workers contributed to memory data for each item in the visual memory task and an average of 31 AMT workers contributed to memory data for each item in the lexical memory task. For the visual memory task, 993 objects have a hit rate and 965 have a false alarm rate. For the lexical memory task, we collected ~100 fewer subjects, and therefore 899 items have a hit rate and 876 have a false alarm rate.

#### Model analysis

In order to address the relationship between visual and semantic image statistics and image memorability, we adopted a linear regression framework. Model diagnostics included overall fit, as well as explicit examination of collinearity across predictor variables (evaluated by the variance inflation factor), and diagnostics on normality (see Figs. 2 and 4).

### Results

The central goal of the current study was to determine what visual and semantic information predicts object memorability. First, we provide regression diagnostics and then summarize the distribution of memory scores on the Visual and Lexical memory tests. We then examine a series of predictors for both memory types based on the visual and semantic features described above. Lastly, we examine interactions between the two forms of memory.

#### Memory performance

**Fig. 7:**
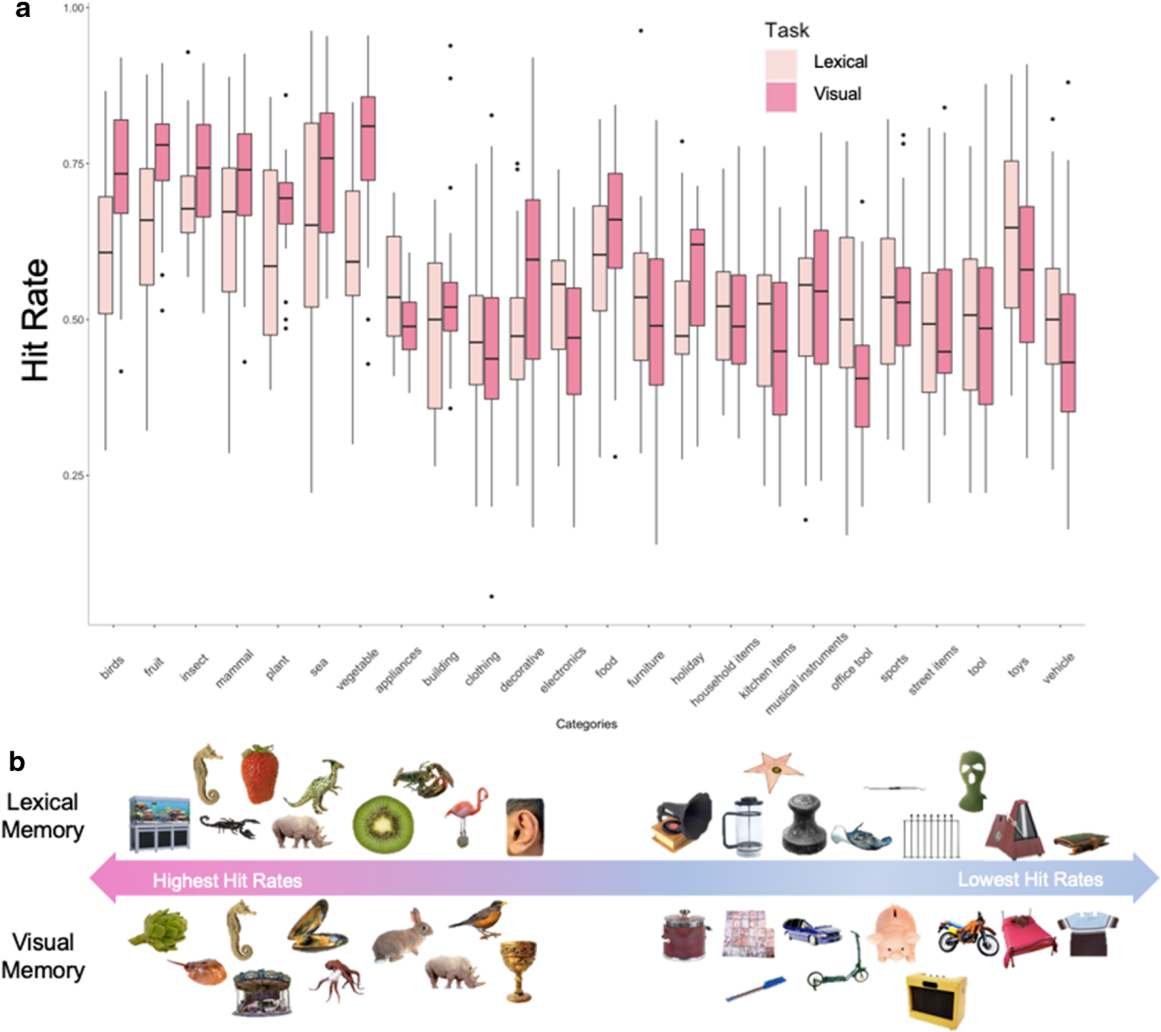
Memory performance summary across category and items. (**A**) Categorical averages of item hit rates for the lexical (light pink) and visual memory task (dark pink). Living items (left side) were considerably better remembered as than non-living items (right side), across all item categories. (Note: categories with fewer than 5 items were removed from this descriptive plot.) (**B**) Visual representation of the most and least memorable object images in the corpus, for both lexical and visual memory

Accuracy for both the visual and the lexical memory tasks on Day 2, as indexed by both Hit Rate (HR) and False Alarm Rate (FAR), as well as response times are shown in Table 2. While the average item-level HRs and FARs are suggestive of low memory performance, the present analyses are focused on what specific factors place individual items along the normative distribution of memory scores. These results, and the interactions between memory tasks and response accuracy, are examined more explicitly below. For descriptive purposes, we also include a summary of the average HRs across item categories (Fig. 7A), which show generally better memory (HRs) for living than non-living categories, but a wide variation across and within each category. We also show the ten most- and least-remembered items (according to HR) within the visual and lexical memory tests, which generally reflects this preference for living categories (Fig. 7B).

**Table 2:** Behavioral performance on visual and lexical memory tests

	Visual memory		Lexical memory	
	M	SD	M	SD
Response accuracy				
Hit rate	0.56	0.18	0.55	0.15
False alarm rate	0.27	0.15	0.44	0.14
Response time (ms)				
Hits	1202	98.48	1288	129.61
Misses	1196	117.78	1304	168.79
Correct rejections	1176	84.09	1318	152.68
False alarms	1245	146.52	1295	290.84

#### Linear models of memorability

##### Regression diagnostics

In this article, simple visual statistics such as JPEG size, proportion non-white space, image energy, and simple semantic statistics such as name agreement, COCA frequency, and number of features are used to assess the stand-alone properties of the objects in the database. The complex semantic statistics we use in this study include *mean distinctiveness* (calculated as 1/[the number of concepts in which a feature occurred]), *correlational strength* (calculated as the average pairwise correlations between a target feature and all other features in the norms), *CSxD* (calculated by taking the slope of the regression line that describes the interaction between correlational strength and distinctiveness), and the entropy of DNN activation values (measure of how distinct objects are across the layers of a DNN). We sought to understand if our semantic and visual models significantly represent the two types of memory we are testing. Our results indicate that memorability of objects is based on various semantic and visual properties, most notably that the more complex statistics better predict memory than item-wise statistics in both the lexical and the visual memory task.

**Fig. 8:**
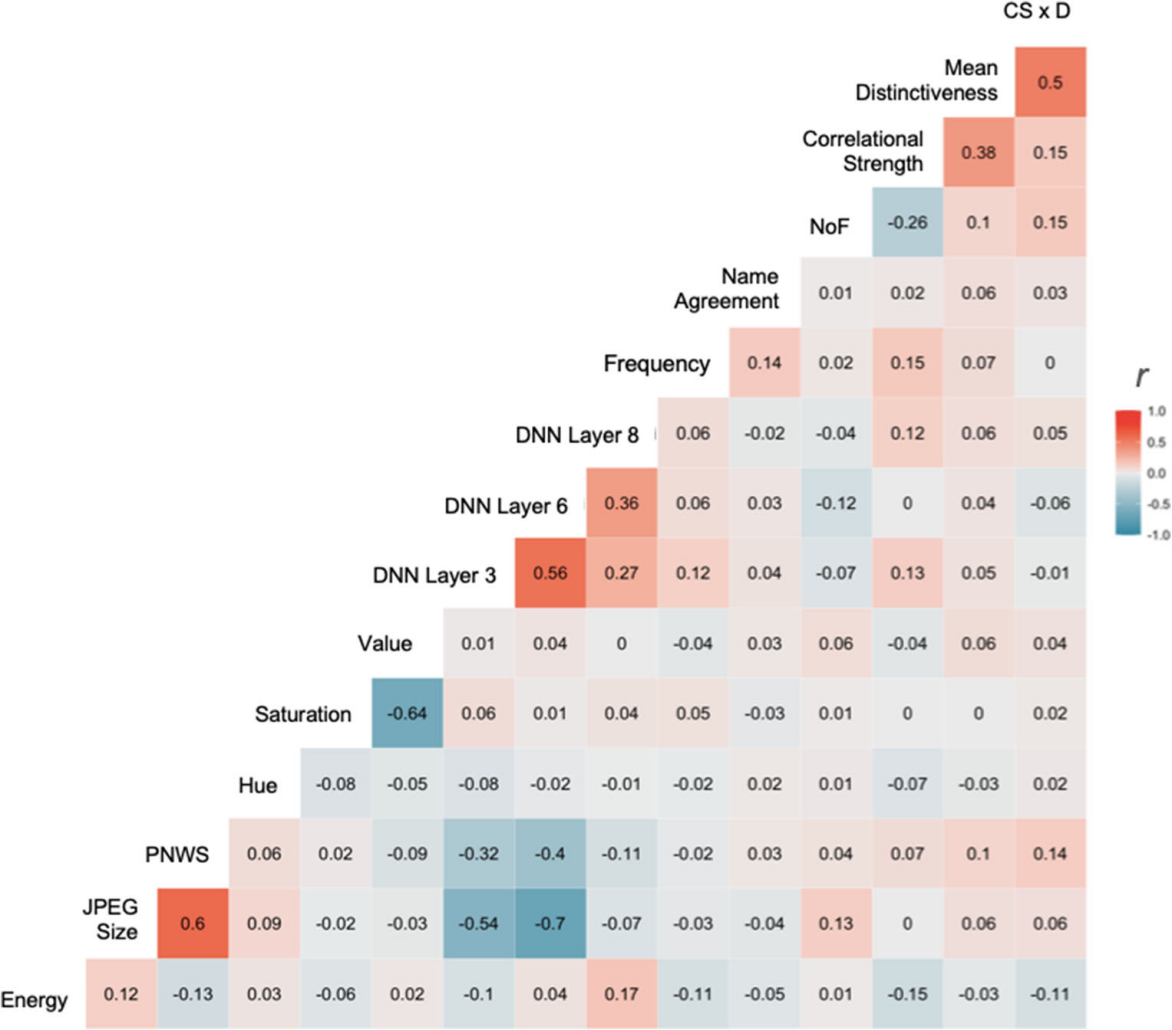
Covariance matrix for all predictors. Covariance across all predictors in the regression model. Variance inflation factor suggests models are not likely to lead to multicollinearity. *NoF* number of non-taxonomic features, *DNN* deep neural network (AlexNet), *CSxD* Correlational Strength x Distinctiveness

In applying regression using large samples of data it is important to be sensitive to the assumptions of the shape of the distribution, including the normality, skew, and kurtosis of the constituent distribution, as well as the collinearity between potential predictors. Model diagnostics describing skew, kurtosis, and normality were conducted on all predictors for all of the regression models and were adjusted and normalized where needed. If a distribution was positively or negatively skewed the appropriate transformation was applied and that value was used in the regression for both the lexical and the visual model. For example, most variables necessitated a log10 transformation, while Hue, Saturation, and Value measures were corrected with a boxcox transformation. Proportion of non-white space, Mean Distinctiveness, and CSxD were considered normal in their original state. Figures 2 and 4 display the original distributions (in pink) and the transformed distributions (in red) for both visual and semantic measures, respectively. To assess collinearity of the predictors in our regression model (see Fig. 8 for covariance across all predictors), the mean of the variance inflation factor (VIF) was calculated for both models. The VIF indicates whether one predictor has a strong linear relationship with another predictor or multiple predictors. It is the ratio of the variance in a model with multiple terms by the variance of the model with only one term. If the VIF is substantially greater than 5 or 10 then the regression model is biased due to multicollinearity (James et al., 2013). The VIF values for all predictors in our model for both false alarm rates and hit rates and for both memory tasks were less than 3.5, suggesting that the models are not likely to lead to multicollinearity.

##### Visual and lexical memory prediction

Here, we first describe the results of the visual properties and their contribution to memory before examining semantic properties. We then report the results of the four separate regression models testing four distinct memory measures: Hit Rates (HRs) and False Alarm Rates (FARs) for both Visual and Lexical memory tests (see Table 3). Before examining the linear regression model using visual and semantic features to predict these scores, we first sought to address the possibility that response bias might lead to an incorrect interpretation of memory scores across items. We calculated criterion item-wise [*c* =  − 0.5(*Z*_*HR*_ + *Z*_*FAR*_)] and found that values for both the visual (mean *c* = 0.25) and lexical tasks (mean *c* = 0.02) were low, suggesting response bias did not play a strong role in the HR and FAR results presented below.

**Table 3:** Regression output

Predictors	Visual memory		Lexical memory	
	Hit rate	False alarm rate	Hit rate	False alarm rate
Visual features				
Energy	**-3.12 (-0.05)**	0.30 (0.005)	-0.60 (-0.009)	0.71 (0.01)
JPEG size	-0.30 (-0.02)	**-3.27 (-0.16)**	**-2.39 (-0.12)**	**-2.45 (-0.13)**
Proportion non-white space	0.43 (0.01)	-0.43 (-0.01)	0.84 (0.02)	-1.19 (-0.04)
Hue	0.57 (0.003)	1.33 (0.006)	0.21 (0.0009)	-0.52 (-0.002)
Saturation	0.28 (0.002)	0.89 (0.005)	1.12 (0.007)	0.69 (0.004)
Value	-1.61 (-0.01)	0.39 (0.002)	1.03 (0.006)	0.21 (0.001)
Early DNN layer (3)	**2.92 (0.29)**	**3.25 (0.28)**	**2.63 (0.23)**	0.13 (0.01)
Middle DNN layer (6)	**-3.14 (-0.45)**	**-5.21 (-0.64)**	**-3.70 (-0.45)**	**-4.14 (-0.51)**
Late DNN layer (8)	**4.03 (0.50)**	**3.25 (0.35)**	0.19 (0.02)	0.97 (0.10)
Semantic features				
Frequency	-0.28 (-0.001)	-0.11 (-0.0005)	**1.93 (0.008)**	**3.72 (0.02)**
Name agreement	**3.76 (0.06)**	**2.03 (0.03)**	**7.99 (0.12)**	**2.87 (0.04)**
Number of non-taxonomic features	-0.20 (-0.01)	-0.29 (-0.02)	0.77 (0.05)	-0.26 (-0.02)
Correlational strength	**7.69 (0.75)**	**5.94 (0.51)**	**5.36 (0.46)**	**2.84 (0.24)**
Mean distinctiveness	**-6.38 (-2.09)**	**-7.32 (-2.08)**	**-5.17 (-1.49)**	**-3.56 (-1.02)**
Correlation x Distinctiveness	**5.48 (0.08)**	**4.24 (0.06)**	**3.85 (0.05)**	**3.58 (0.05)**

*Note.* The table represents t-values (beta values in parenthesis). Bold font indicates significance, p < 0.05. Adjusted R^2^ for hit rates for the visual memory = 0.16 and for lexical memory = 0.14. Adjusted R^2^ for false alarm rates for visual memory = 0.12 and for lexical memory = 0.07

First, examining the capacity for visual properties to predict later memory, we found that entropy values based on activation of all layers of the DNN (Early, Middle, and Late based on AlexNet Layers 3/6/8, respectively), had a significant influence on nearly all measures of memory discriminability. Early DNN information was a significant predictor of memory in both tasks. This layer, which is organized roughly by shape (see Fig. 3A) significantly predicted HRs in the lexical (*β* = 0.23, t = 2.63, p = 0.009) and visual memory tasks (*β* = 0.29, t = 2.92, p = 0.004); FARs were also influenced by this early visual information in the visual (*β* = 0.28, t = 3.25, p = 0.001) but not the lexical memory task (*β* = 0.01, t = 0.13, p = 0.90). Middle DNN information, which is roughly organized by color and orientation information, significantly predicted all memory measures (in the opposite direction) for both the visual (HR: *β* = -0.45, t = -3.14, p = 0.002; FAR: *β* = -0.64, t = -5.21, p < 0.000001) and the lexical memory tests (HR: *β* = -0.45, t = -3.70, p = 0.0002; FAR: *β* = -0.51, t = -4.14, p = 0.00004). Late DNN information, which is roughly organized by category (which we would predict from the last layer of a DNN), significantly predicts memory in the visual memory task (HR: *β* = 0.50, t = 4.03, p = 0.00006; FAR: *β* = 0.35, t = 3.25, p = 0.001), but not the lexical memory task (HR: *β* = 0.02, t = 0.19, p = 0.85; FAR: *β* = 0.10, t = 0.97, p = 0.33). Such a pattern of findings suggests strong evidence that visual memory is reliant on a continuum of complex image properties, but that Lexical memory may generally be less reliant on these properties.

In contrast to the more complex values derived from the DNN, we found only a few other visual properties to be predictive of memory success. Simple image statistics such as mean hue of an image was not predictive of memory in either the visual (HR: *β* = 0.003, t = 0.57, p = 0.57) or the lexical memory task (HR: *β* = 0.0009, t = 0.21 p = 0.83). Mean saturation was also not predictive of memory in either the visual (HR: *β* = 0.002, t = 0.28, p = 0.78) or the lexical (HR: *β* = 0.007, t = 1.12, p = 0.26) memory task. Mean color value was not predictive of memory in the visual memory task (HR: *β* = -0.01, t = -1.61, p = 0.11) or the lexical memory task (HR: *β* = 0.007, t = 1.03, p = 0.30). These results indicate that simple properties describing color features of images are not memorable, regardless of the task. This result is in line with other findings on the ability of basic image properties to predict memory (Dubey et al., 2015). Image energy (a measure derived from the average spectral power of an image; for more information see Torralba & Oliva, 2003) negatively predicted HR in the visual memory task (*β* = -0.05, t = -3.12, p = 0.002), but not the lexical memory task (*β* = -0.009, t = -0.60, p = 0.76). In addition, JPEG size (a measure of image complexity) offered a significant negative prediction for FARs in both the visual (*β* = -0.16, t = -3.27, p = 0.001) and the lexical (*β* = -0.13, t = -2.45, p = 0.01) memory tasks. JPEG size *did not* predict memory in the visual (HR: *β* = -0.02, t = -0.30, p = 0.77) memory task, but it did predict memory in the lexical task (HR: *β* = -0.12, t = -2.39, p = 0.02). This result is intriguing because it suggests that image complexity serves to make an object less confusable with other objects, or, conversely, that simpler images are generalized across mnemonic exemplars. No other image statistic has a significant influence on any memory measure (see Table 3). Overall, these results suggest that simple image statistics play a generally small role in predicting memory for objects (especially with regard to semantic properties discussed below).

Turning to our semantic properties, we found that in the visual memory task, the complex feature-based predictors generally proved to be stronger than more simple statistics as predictors of later memory strength. Correlational strength was a strong predictor of both item-wise HRs (*β* = 0.75, t = 7.69, p < 0.000001) and FARs (*β* = 0.51, t = 5.94, p < 0.000001) in the visual memory task. Such a result is intuitive assuming that when attempting to make a recognition judgment on an image, participants reactivated co-occurring features for an object to ease conceptual processing, as is assumed during object recognition (Taylor et al., 2011). In the lexical memory task, correlational strength predicted item-wise HRs (*β* = 0.46, t = 5.36, p < 0.000001), and, to a significant but weaker extent, FAR (*β* = 0.24, t = 2.84, p = 0.005). Viewing an image and attempting to recognize it by viewing its corresponding lexical label relies on recall of both visual features of that object, as well as descriptive features of that object (Taylor et al., 2012). Mean distinctiveness also influenced the visual memory HRs (*β* = -2.09 t = -6.38, p <0.000001) and FARs (*β* = -2.08, t = -7.32, p < 0.000001), as well as lexical memory HRs (*β* = -1.49, t = -5.17, p < 0.000001) and FARs (*β* = -1.02, t = -3.56, p = 0.0004). Interestingly, mean distinctiveness predicted memory HRs in the *opposite* direction from CS, such that objects with more distinctive features are associated with lower hit rates. A ready alternative explanation for this effect is that objects with shared, highly correlated properties – i.e., typically animals and other living items – will be better remembered. While it is intuitive to see how more correlated features may lead to better memory for an object due to a greater number of interrelated cues, it may seem less intuitive with respect to MD. Distinctive features are thought to ease activation of a unique representation of an object (Biederman & Kalocsai, 1997; Clarke & Tyler, 2015; Martin et al., 2018); however, in our data it is possible that while the distinctiveness of the item features facilitate identification, retention in memory suffers from highly distinctive features. Subsequent mediation analyses below cast doubt on the possibility that this negative weight is simply a product of interactions with other semantic (or visual) predictors, as this negative relationship is present in both the *a* and *b* effects in the mediation.

One direct means of disentangling this somewhat perplexing result is in an examination of a third CSA statistic. In addition to CS and MD, the mean correlational x distinctiveness (CSxD, also known as “slope”) measure was a positive predictor of both the visual (HR: *β* = 0.08, t = 5.48, p <0.000001; FAR: *β* = 0.06, t = 4.24, p = 0.00003) and the lexical (HR: *β* = 0.05, t 3.85, p = 0.0001; FAR: *β* = 0.05, t = 3.58, p = 0.0004) memory tasks. These results indicate that memory for objects with highly correlated distinctive features (as is the case in many living items; see Taylor et al., 2012) is better in both the visual and the lexical memory tasks. Distinctive features on their own might not be beneficial for memory, but if features are highly correlated (e.g., animals that have eyes, ears, nose) *and* highly distinctive (e.g., animals that have wings and feathers) they may contribute to overall memory for that object.

In addition to these semantic predictors, COCA frequency and name agreement also predicted memory. COCA frequency of a concept positively predicted both lexical HRs and FARs, (HRs: *β* = 0.008, t = 1.93, p = 0.05; FAR: *β* = 0.02, t = 3.72, p = 0.0002), in keeping with the notion that high-frequency items are more likely to be endorsed as “old” (Badham et al., 2017); no effect of Frequency was seen for visual memory. Name agreement was predictive of memory in the visual memory task (HRs: *β* = 0.06, t = 3.76, p = 0.0002; FAR: *β* = 0.03, t = 2.03, p = 0.04) and the lexical memory task (HR: *β* = 0.12, t = 7.99, p < 0.00001; FAR: *β* = 0.04, t = 2.87, p = 0.004). Knowing the name of an object is clearly necessary to recall an item given the cue presented in the lexical retrieval task, but this information may also be helpful when recognizing an image. Nonetheless, the low name agreement in roughly one-quarter of our sample (276/995 items with < 50% name agreement), while typical for everyday concept norms such as these (Devereux et al., 2014), nonetheless suggests that some “miss” responses could have been driven by not recognizing the specific lexical cue used in our study. Further work may be necessary with more constrained item stimuli sets. In an effort to encourage such exploration, our dataset has a range of name agreement values and users of our dataset are free to access these data and determine appropriate cut off points.

Lastly, we sought to further address the possibility that response bias may have influenced item-wise memory scores, given that the values for many predictors of both HR and FAR trend in the same direction (see Table 3). While criterion scores were generally low (see above), we found that using d’ as our measure of memory did attenuate predictors on both the visual and lexical tasks compared to HRs and FARs, such that for the visual memory model, Image Energy (t = -2.54, p = 0.01), JPEG size (t = 2.33, p = 0.02), and CSxD (t = 2.43, p = 0.02) remained as significant predictors, while for the lexical memory, Name Agreement (t = 5.34, p = 0.000002), CS (t = 3.11, p = 0.002), and MD (t = -2.22, p = 0.03) remained significant. All other predictors in these models were not significant. This finding suggests that measures of response bias and memorability beyond hit rate should be considered when estimating an item’s intrinsic memorability.

#### Visual–lexical memory relationships

**Fig. 9:**
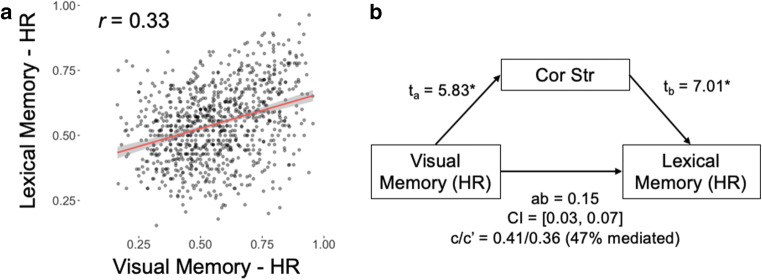
Relationships between visual and lexical memory. (**A**) Correlations between hit rates (HR, above) for visual and lexical memory scores. (**B**) Mediation analyses demonstrate that correlational Strength helps to explain a significant proportion of the covariance between our memory test HRs

Lastly, in order to test the independence of visual and lexical memory, we performed two additional analyses on these data. First, we assessed the relationship between visual and lexical memory at the level of items using Pearson’s correlations. As shown by Fig. 9A, we found that Hit Rates for the two tests were significantly related (r_993_ = 0.33, p < 0.0001). FARs, while also correlated (r_993_ = 0.54, p < 0.0001), were not examined, due to the fact that many distinct confounds could explain such errors in memory. This relationship therefore engenders the question, “*what common qualities of a retrieval stimulus make an item memorable across perceptual and conceptual memory retrieval conditions?*”

To answer this question, we explored the relationship between visual and lexical memory hit rates using mediation analyses, which helps to explore potential sources of variation mediating an observed relationship between a predictor and outcome variable. In our analysis, we posited the significant predictors in the regression output above (see Table 3) as mediators of the relationship between lexical and visual memory hit rates, where mean hit rates were treated as item-level observations (see Table 4). As such, we expect that the observed relationship between the predictor (visual HR) and outcome variables (lexical HR) could be at least partially explained by an underlying visual or semantic property. Of these, only Correlational Strength (i.e., the measure of relatedness of items’ constituent features) acted as a significant mediator of the relationship between visual and lexical memory hit rates (*ab* = 0.15, CI = [0.03, 0.07], 47% mediation; see Fig. 9B). Unsurprisingly, CS showed strong relationships with both visual and lexical memory HRs (*a* and *b* effects in this mediation, respectively). Collectively this result suggests that the underlying similarity in item memorability across our two tests could be at least partially explained by the underlying semantic structure common to perceptual and conceptual representations of a given item.

**Table 4:** Mediation between visual and lexical memory hit rates

Mediator	a	b	c’	ab	R^2^	CI
HRs; total effect (c) = 0.41, t = 11.2***						
Visual predictors						
Early DNN	0.11 (3.03)	0.06 (1.86)	0.40 (11.00)	0.01	0.12	[0, 0.02]
Middle DNN	-0.08 (-1.52)	-0.04 (-1.62)	0.40 (11.14)	0.00	0.11	[0, 0.01]
Late DNN	-0.03 (-0.55)	0.10 (4.09)	0.41 (11.38)	0.00	0.13	[-0.02, 0.01]
Semantic predictors						
CS	0.03 (5.83)	1.52 (7.01)	0.36 (10.02)	0.05	0.15	[0.03, 0.07]
MD	0.01 (0.92)	-0.06 (-0.24)	0.40 (11.18)	0.00	0.11	[0, 0]
CSxD	0.46 (2.40)	0.03 (4.21)	0.39 (10.96)	0.01	0.13	[0, 0.03]

*Note.* For all models, df = 992. HR = hit rate.

*FAR* false alarm rate, *DNN* deep convolutional neural network, *CS* correlational strength, *MD* mean distinctiveness, *CSxD* interaction between CS and MD

## General discussion

The current analysis presents a large-scale database of object concepts with a comprehensive assessment of visual and semantic properties, as well as visual and lexical memorability information based on independent tests of object memory. In our analysis, we identified two critical observations that help to guide future analyses of object memory. First, we found that complex visual and semantic features (e.g., DNN-based entropy, correlational strength of features) were strong predictors of item memory for both visual and lexical tests, a finding that highlights the importance of considering these fine-grained relationships between items in stimuli selection. Second, we found that despite the fact that visual and lexical item memory had a relatively modest correlation in hit rates across items, performance was driven by similar object properties, and that correlational strength helped to explain the relationship between these memory scores. This result suggests that object representations have distinct visual and verbal components, but remain grounded in the same core substrate. Furthermore, this suggests that memorability may not be an intrinsic quality of an image but may instead depend on the context under which the memory for such image is tested.

### Visual and semantic feature statistics

A principal contribution of the current article is a highly dimensional image corpus comprising of 995 concrete objects spanning a wide range of different categories, along with visual and semantic features and output from the associated memory study, all of which are available within a centralized database (http://mariamh.shinyapps.io/dinolabobjects). Within this database are a variety of object statistics that range from common to unique (e.g., lexical frequency and feature-based stats), visual to semantic (image energy and correlational strength), simple to complex (e.g., JPEG size and DNN information). Furthermore, this database complements other rich concept corpuses (Devereux et al., 2014; Hebart et al., 2019; Ken McRae et al., 2005) by including statistics on the two different kinds of memory for each object image. This may serve as a useful heuristic for selecting object stimuli for experiments with patients experiencing age-related memory disorders (Bainbridge et al., 2019). The statistics we have compiled allow integration of object concepts along several interrelated schemes, such that one can use these data, for example, to visualize how concept categories are organized according to semantic feature information (Fig. 3D), or how DNN organization draws upon more basic image properties like RGB balance (Fig. 3A). All data are publicly available, including the images, the visual and semantic properties of the images, as well as the memory scores (both HRs and FARs) established on the separate tests of memory based on visual and lexical cues.

In the visual domain, we contribute a rich form of visual statistics based on three separate layers of a deep convolutional neural network. There is evidence that DNNs share some similar properties to the ventral visual pathway (Cichy et al., 2016), and can even surpass traditional theoretical models. In the present study, we sought to understand the dimensionality of visual information in our object images using activation values of the DNN in order to calculate entropy of the an early, middle, and late layer of interest (Layers 3, 6, and 8). One consistent pattern from the memory prediction models above (Table 3) is that while early DNN information contributed to both visual and lexical memory, late-layer DNN information predicted memory in the visual memory task, but not the lexical memory task. In addition, DNNs are trained to classify objects based only on visual information, not semantic information, and therefore its total value in predicting memory based on verbal cues is limited. While visual DNNs can appropriately label images, they do not capture the semantic relationship between items. For example, an orange and a banana are semantically more related (i.e., both are fruits) than an orange and a basketball (i.e., more visually similar). The notion that visual similarity requires semantic similarity is an important feature of the ventral visual processing stream (Devereux et al., 2018), which is not currently captured through visual DNNs. This emaphasizes the importance of considering semantic statistics from large property-norming studies such as the one introduced here.

### The role of complex semantic properties in predicting memory

A major finding from this analysis is that semantic feature statistics associated with complex semantic properties, based on the interrelatedness of the constituent features of items (e.g., mean distinctiveness, correlational strength), were significant predictors of hit rates on the visual and lexical memory tests. This novel finding demonstrates that feature statistics that describe the interrelatedness of constituent features across items have a strong mnemonic value for both perceptual and conceptual memory. The direction of this prediction was intuitive when one places a premium on interrelatedness. Correlational strength had a strong positive prediction score (Table 3), suggesting that highly correlated features may provide a richer set of cues from which to encode and retrieve specific object concepts or images. In contrast, the mean distinctiveness of features in turn had a strong *negative* prediction score, a finding that seems somewhat counterintuitive if one places a premium on item frequency or typicality as a strong mnemonic predictor for episodic memory judgments (Fernandez et al., 1998; Schmidt, 1996). Helping to disambiguate this result, we also found that a third measure (CSxD) had a unique and positive prediction accuracy for hit rates in both Visual and Lexical memory tasks, suggesting distinctive features do help with an object’s memorability, but only when those distinctive features are considered within a broader semantic network of features (Taylor et al., 2012).

Visual working-memory and episodic memory research are both based on the premise that objects are represented as bound units (Brady et al., 2013; Gajewski & Brockmole, 2006; Luck & Vogel, 1997; Morey & Cowan, 2005). While both forms of memory are dependent on the same high-level object representations, they do not account for discrepancies in memory errors for objects with the same features. Thus, while certain visual and semantic traits explain the memorability of scenes (e.g., spatial layout of a scene, as in Isola et al., 2014), and faces (e.g., atypicality of a faces, as in Bainbridge et al., 2013), they do not capture all of the variance in memorability scores across all items within a corpus. Accordingly, these approaches have rarely found a strong role for semantic content in predicting item memorability. The current data present some evidence that one of the keys to understanding this variability in the intrinsic memorability of an item lies in considering the variance in properties such as MD, CS, or CSxD.

These meaningful properties have been shown to be predictive of memory in other studies. In Brady et al. (2013), participants were shown real-world objects and given a forced-choice color and state (e.g., some meaningful manipulation on an object’s functional or observable properties) test after a short or long delay. The results indicated that participants’ ability to detect color decreased over time, whereas their ability to detect state remained stable across short and long delays. This suggests that while salient visual features like color or luminance are forgotten quickly, meaningful properties that are integrated with object representation are retained longer. The problem with previous approaches is that “meaningful” or “semantic” is often defined in terms of discrete categories or cardinal attributes, and not a continuous metric amenable to rigorous analysis. Furthermore, our analysis helps to bridge the gap between complex semantic and object memorability literatures, and thus offers some clear predictions on how feature relatedness will affect performance on a given task.

### Item memory differs between visual and lexical tests

Our second finding showed that similar item characteristics predict memory on both tests, and that visual and lexical hit rates were correlated across items. This finding supports the idea that the organization of perceptual and conceptual memory traces share similar underlying item characteristics, but nonetheless draw on a number of unique representational forms based on the modality of retrieval (Saffran et al., 2003). As shown in Fig. 9A, hit rates on the two tests demonstrated a modest correlation (r_784_ = 0.33), an effect size that is moderate given the number of observations (n = 784). This inflation due to sample size was further evidenced by examining the same cross-test relationship within each category, and also found no significant item-wise relationship between hit rates in the two tests (all rs < 0.2 within each category). Nonetheless results from our memory prediction analyses suggested that both visual and lexical memory performance depended on similar object properties.

What do these findings mean for the study of visual memory of everyday things? Our data show strong support for the idea that the fidelity of long-term memory representations is driven by complex semantic properties that describe the structure of an object’s constituent features. Such a result provides an item-wise perspective on a range of previous studies showing that, compared to perceptual information, semantic information can have stronger influences on both hit rates and false alarm rates. For example, the fact that false alarm rates were predicted by all three complex feature-based semantic statistics (MD, CS, and CSxD; see Table 3) provides greater detail to the finding that pre-existing conceptual information detracts from the processing of perceptual, item-specific features (Konkle et al., 2010b; Koutstaal et al., 2003). These earlier studies were often based on a rather general notion of semantic relatedness, category membership, whether for real-world (Brady et al., 2008; Konkle et al., 2010a) or novel objects (Hout & Goldinger, 2010; Koutstaal et al., 2003), rather than a detailed set of semantic properties as presented in the current study. Category members may vary widely in their relatedness – and hence, how strongly a category exemplar may act as a potential memory lure. Our more detailed semantic feature information helps to quantify this variation across object exemplars within a category, and may serve as a principled basis on which to explicitly examine the effect of semantic relatedness on false memory. Thus, the current dataset provides greater detail on why semantic relatedness influences broad category relationships.

Our article attempts to bridge the object perception literature (which focuses on item-wise properties) and the episodic memory literature (which focuses on subject-level performance). Given our interest in measuring memory for items, we averaged the frequency of hits (1) and misses (0) across participants in both the visual and the lexical memory tasks and conducted a linear regression model. However, analyses quantifying subject-level bias towards different object classes or categories might identify not only the degree to which a particular object is memorable across contexts, but also the capacity for biased memory endorsements. For example, more familiar items may be seen as more memorable (Nickerson & Adams, 1979). More broadly, these data speak to the necessity of considering the concept of image memorability as a potentially malleable property across individuals and test types, and we release our data via public repositories to encourage such exploration.

Lastly, our mediation analyses (Fig. 9B) lend support to the idea that greater correlational strength facilitates conceptual processing (Clarke et al., 2013; Cree & McRae, 2003; Devereux et al., 2016). These results clearly show a relevance to the relative independence of perceptual and conceptual representations. As the current analysis was focused on item-wise characteristics in explaining this independence, we performed a number of follow-up tests using these item-wise characteristics to help explain this effect. We tested multiple mediators of the visual-lexical memory relationship, namely predictors that were identified in our item-wise regression analyses as significant predictors of either visual or lexical memory performance. As expected, only semantic features showed a significant mediation of the relationship between visual and lexical memory, while no visual feature demonstrated a significant mediation. Nonetheless, it is possible that the prominent role for complex semantic features in predicting both forms of memory may be driven by the fact that the encoding task required a semantic (living, non-living) judgment, thus focusing participants on semantic properties of the object image before them. Future studies may benefit from a more explicit comparison of encoding strategies designed to highlight complimentary feature types (e.g., Koutstaal et al., 1999); we have endeavored to make our dataset and associated code readily available to facilitate this work.

## Conclusion

In addition to furthering our understanding of the influence of different stimulus properties on item-wise memorability of object images, the current results also showcase how broad, comprehensive image databases can be utilized to answer fundamental questions on the nature of memory representations. The current study goes beyond memorability studies that examined a single memory task (Isola et al., 2014), and establishes a bridge between this work focused on *stimulus* factors, and those that focus on memory *processes*. Furthermore, correcting for possible response bias (by using d’) did attenuate some of the effects, such that Image Energy, JPEG size and CSxD remained significant predictors in the visual memory task, while Name Agreement, CS, and MD remained significant for the lexical memory task. Thus, while both forms of memory are supported by complex semantic properties, these results do suggest that the predictive properties for image memorability begin to diverge somewhat depending on how memory is tested, and fight against the idea that image memorability is an intrinsic property of an object image. While HR and FAR are the primary focus of the paper, such a finding highlights the necessity to consider latent response bias, the potential for objects to act as conceptual lures, or other mnemonic factors that suggest the concept of memorability should be defined beyond hit rates alone. By investigating object memory with two different memory tests, we show that there is no single set of “memorability features” that can predict memory for objects in all scenarios; rather these scores are more likely based on the context (i.e., the type of memory test) in which the stimuli are tested. As such, future studies investigating object memory can take into consideration the transfer appropriate processing principle and test stimuli in multiple memory paradigms. We hope that this new set of object property norms and their associated memory strength in multiple domains will help to foster new investigations on object representation and the fidelity of object memory.
